# Non-pharmacological interventions to support return to education and work for adolescent and young adults (AYAs) following a cancer diagnosis: a scoping review protocol

**DOI:** 10.12688/hrbopenres.14096.2

**Published:** 2026-01-16

**Authors:** Naomi Algeo, David Mockler, Stacey Braddish, Matthew Barrington, Niamh O'Sullivan, Joy Lewis, Scheryll Alken, Peter McCarthy, Joanne O'Mahony, Kathleen Bennett, Claire McCall, Amy Nolan, Catherine Buckley, Sonya Collier, Louise Mullen, Nina Orfali, Aoife O'Gorman, Ruth McMenamin, Denise Dockery, Nickola Pallin, Deirdre Connolly

**Affiliations:** 1 Discipline of Occupational Therapy, Trinity College Dublin School of Medicine, Trinity Centre for Health Sciences, St. James's Hospital, Dublin, Ireland; 2 Trinity St. James's Cancer Institute, St James's Hospital, St. James's Hospital, Dublin, Ireland; 3 Trinity College Dublin School of Medicine, Trinity Centre for Health Sciences, St. James's Hospital, Dublin, Ireland; 4 Patient and Public Involvement Representative, Dublin, Ireland; 5 CHI at Crumlin, Children's Health Ireland, Cooley Road, Crumlin, Dublin, Ireland; 6 Data Science Centre, Royal College of Surgeons in Ireland Division of Population Health Sciences, Dublin, Ireland; 7 Children, Adolescents and Young Adults (CAYA), Irish Cancer Society, Dublin, Ireland; 8 National Cancer Control Programme, Health Service Executive, Dublin, Ireland

**Keywords:** cancer survivorship; employment; quality of life; reintegration; schooling

## Abstract

**Objective:**

This scoping review will explore and chart the evidence relating to non-pharmacological interventions that support education and/or employment for adolescent and young adults (AYAs) following a cancer diagnosis.

**Introduction:**

The number of newly diagnosed AYAs with cancer is increasing and so too is the number of AYAs living with and beyond cancer. In line with missed schooling and long-term side-effects of treatment, it is unsurprising that this group may under-perform academically and be at higher risk of unemployment in comparison to their peers. Developing an overview of existing interventions to support education and/or employment for this cohort, is therefore important.

**Inclusion Criteria:**

Studies that explore non-pharmacological interventions that support education and/or employment for AYAs (aged 15–39 years at cancer diagnosis) post-cancer diagnosis. Interventions can be group-based, individual, and/or online in format, and can be vocational, psychosocial, physical, educational or multidisciplinary in nature. Qualitative, quantitative, mixed methods studies, case studies, observational studies, reports and theses will be included.

**Methods:**

This scoping review will follow the Joanna Briggs Institute (JBI) methodology for scoping reviews. Databases to be searched include EMBASE, Web of Science, Medline (OVID), CINAHL, and PsycInfo, with no limitation on publication date. Grey literature will be searched, limited to the first 100 searches on Google Scholar. Titles and abstracts will be screened and two independent reviewers will review identified fill-texts. A data extraction tool will be used for data extraction.

## Introduction

Internationally, the considered age range of Adolescent and Young Adult (AYA) cancer survivors is 15–39 years (at diagnosis) ( Desandes & Stark, 2016). The number of newly diagnosed AYA cancers is increasing and so too is the number of AYA living with and beyond cancer, resulting in heightened focus on survivorship needs. This recent paradigm shift in survivorship care has focused on optimising quality of life for those living with and beyond cancer, including reintegration into education and employment ( Vaz-Luis *et al.*, 2022).

Physical and psychosocial sequalae experienced by AYA cancer survivors during and after cancer treatment include fatigue, cognitive dysfunction, anxiety, and depression ( Baker & Syrjala, 2018; Ikonomidou, 2018; LaRosa *et al.*, 2017; Lea *et al.*, 2020; Mittal *et al.*, 2022; van Deuren *et al.*, 2020; Zebrack, 2011). It is commonly reported that this cohort will face ‘overwhelming’ physical and cognitive demands on their re-entry to education and employment ( Brauer *et al.*, 2017). Challenges associated with education reintegration include expedited return to keep up with peers, limited training for educators on how to best support reintegration, and the impact of altered body image during this adolescent stage ( Brauer *et al.*, 2017; Fardell *et al.*, 2018; Thompson *et al.*, 2015). While there remains partial overlap of physical and psychosocial barriers to employment reintegration for AYAs, other factors can influence this transition including returning to dependency on parents, fear of potential workplace discrimination, and workplace accommodations ( Fardell *et al.*, 2018; Husson *et al.*, 2018; Ketterl *et al.*, 2019), although these can vary depending on societal context (e.g., country-specific legislation/entitlements).

In line with missed schooling and long-term side-effects of treatment, it is unsurprising that this group may under-perform academically in comparison to their peers, which in turn can lead to employment and financial difficulties in later life ( Elsbernd *et al.*, 2018). Furthermore, an international report for survivors of children, adolescents and young adults (CAYA) cancer highlighted evidence that this cohort are at higher risk of lower educational achievement and unemployment in later life ( Devine *et al.*, 2022). Return to education and employment is therefore often cited as challenging by AYA cancer survivors ( Barrett *et al.*, 2018; Ikonomidou, 2018).

The European Code of Cancer recently identified the right of all cancer survivors to fully resume educational, economic, and social roles ( Lawler *et al.*, 2021). However, a gap in current research includes education and employment reintegration, where AYAs self-identified research on return to education and work as a top priority in a UK research priority-setting exercise ( Aldiss *et al.*, 2019). This has also been recognised internationally where the need to focus on education and career development for AYA cancer survivors has been cited as a core priority ( Altherr *et al.*, 2023; National Cancer Control Programme, 2022). Therefore, supports to empower AYAs who have had cancer to stay or return to education and/or work is an important issue for both AYAs and society. With the view to developing education and work-related supports for AYAs with cancer in the Republic of Ireland, a scoping review of relevant interventions in relation to type, content, evaluation and impact, will be completed.

## Methods

The proposed scoping review will be conducted in accordance with the Joanna Briggs Institute (JBI) methodology for scoping reviews ( Peters *et al.*, 2020). This scoping review protocol was registered on the Open Science Framework (OSF) database on 21 ^st^ February 2025 and reporting will be guided in accordance with the Preferred Reporting Items for Systematic Reviews and Meta-Analyses Protocols (PRISMA-P) statement ( Moher *et al.*, 2015). This protocol will be conducted in accordance with the JBI methodology for scoping reviews to ensure a systematic methodology that can be replicated. In addition, the scoping review framework proposed by Arksey and O’Malley (2005) is used to structure this protocol, which suggests five steps: (i) identifying the research question, (ii) identifying relevant studies, (iii) selecting studies, (iv) charting the data, and (v) collating, summarising, analysing, and presenting results. The planned scoping review will be reported in accordance with the PRISMA Extension for Scoping Reviews (PRISMA-ScR) ( Tricco *et al.*, 2018).

### Stage one: Identifying the research question

The main research question, guided using the Population-Concept-Context framework ( Peters *et al.*, 2020), is: What is the available evidence regarding non-pharmacological interventions that support education and/or employment outcomes for AYAs? In this instance, population refers to AYAs who were diagnosed with cancer (diagnosed aged 15–39 years inclusive) who have had a cancer diagnosis, context refers to interventions that support education and/or employment outcomes for AYAs, and context refers to any setting (acute/primary care/workplace/school/etc.) in any geographical location. The scoping review protocol has been reviewed by a patient and public involvement representative (who is also a co-author) on the research steering committee who has also informed objectives of this review.

The primary aim of this scoping review is to identify and summarise the core elements (i.e., content, delivery, resources, length, and format) of non-pharmacological interventions/supports for adolescents and young adults who have had a cancer diagnosis to support return to education and/or work.

Secondary objectives include:

1. To identify and summarise measures used to test the impact of these type of interventions on education and/or work and other outcomes.
2. To identify the most commonly used theoretical frameworks underpinning programmes that support return to education and/or work for AYAs.
3. To identify and describe the range of outcomes reported for non-pharmacological interventions and support programmes/services that support return to education and/or employment for AYAs.

The aim of this scoping review is to explore and chart the evidence relating to non-pharmacological interventions that support education and/or employment for AYAs.

*Eligibility criteria:* Inclusion criteria will be limited to peer-reviewed and non-peer reviewed studies and programmes that focus on interventions/programmes that support education and/or employment (concept) for AYAs after a cancer diagnosis (population), across any setting (acute/primary care/workplace/school/etc.) (context) ( Table 1). This scoping review will consider both experimental and quasi-experimental study designs including randomised control trials, non-randomised control trials, before and after studies, and interrupted time series studies. In addition, analytical observational studies including prospective and retrospective cohort studies, case-control studies and analytical cross-sectional studies will be considered for inclusion. This review will also consider descriptive observational study designs, including case series, individual case reports, and descriptive cross-sectional studies for inclusion. Qualitative studies will also be considered that focus on qualitative data including, but not limited to, designs such as phenomenology, grounded theory, ethnography, qualitative description, and action research. Systematic reviews that meet the inclusion criteria will also be considered, depending on the research question. Text and opinion papers will also be considered for inclusion in this scoping review. While some study designs (e.g., cross-sectional, qualitative, etc.) do not allow assessment of intervention effectiveness over time, they may provide valuable information on the types of interventions offered to AYAs and the range of outcomes measured. Therefore, we will include these studies to capture descriptive data on interventions and outcomes.

**Table 1.** Eligibility Criteria.

Category	Inclusion Criteria
Population	• Adolescents and young adults (AYAs) aged **15–39 years** at time of cancer diagnosis • Age range selected to reflect internationally recognised definitions (e.g., Desandes & Stark, 2016). • Studies including AYAs during or after cancer treatment.
Concept	• **Non-pharmacological interventions** designed to support **education and/or employment** outcomes for AYAs. • Interventions may be group-based, individual, online, vocational, psychosocial, physical, educational, or multidisciplinary. • Outcomes may include school reintegration (e.g., e.g., binary yes/no for education status, academic performance, etc.), return-to-work (e.g., binary yes/no for working status; work satisfactions etc.), or employability.
Context	• Any setting, including acute care, primary care, community, workplace, or school. • Studies from any geographical region.
Types of Evidence Sources	**Peer-reviewed and non–peer-reviewed sources**, including: **Experimental and quasi-experimental:** • Randomised controlled trials • Non-randomised controlled trials • Before-and-after studies • Interrupted time series **Analytical observational:** • Prospective and retrospective cohort studies • Case–control studies • Analytical cross-sectional studies **Descriptive observational:** • Case series • Case reports • Descriptive cross-sectional studies **Qualitative research:** • Phenomenology, grounded theory, ethnography, qualitative description, action research, etc. **Other sources:** • Systematic reviews (where relevant) • Text and opinion papers • Programme descriptions or evaluations

*Population:* This scoping review will consider studies of non-pharmacological interventions and support programmes that include AYAs who were diagnosed with a cancer diagnosis (aged 15–39 years inclusive). The age range considered for AYAs can vary from country to country. For example, in the Republic of Ireland, the recognised age range for this cohort is 16–24 years ( Alken *et al.*, 2020). However, for the purpose of this review and to yield a breadth of supports, the age range of 15–39 years which is recognised internationally will be considered ( Desandes & Stark, 2016). Intervention eligibility will only be considered if age range falls within 15–39 years (e.g. study focusing on ages 12–24 would be excluded).

*Concept:* The proposed scoping review is designed to explore non-pharmacological interventions that support education and/or employment outcomes for AYAs. Interventions can be group-based, individual, and/or online in format, and can be vocational, psychosocial, physical, educational or multidisciplinary in nature. Pharmacological or medical interventions are excluded, as the focus of this review is on behavioural, psychosocial, vocational, educational, and rehabilitation-oriented strategies. From here on in, we will refer to these approaches simply as interventions.

*Context:* The aim of this scoping review is to explore the breadth of current literature published on interventions that measure education and/or employment outcomes for AYAs. Therefore, studies conducted in any setting (acute/primary care/workplace/school/etc.) in any geographical location will be considered.

*Patient and Public Involvement:* A patient and public involvement (PPI) contributor, who is also a co-author on this protocol (SB), was involved in the development of the review objectives, refinement of the research question, and identification of key concepts relevant to AYAs following a cancer diagnosis. The PPI member provided feedback on the scope of the review, the relevance of outcomes relating to education and employment, and contributed to ensuring that the planned review reflects patient priorities. No additional consultation stage is planned beyond their ongoing involvement in interpreting findings during the analysis and dissemination stages.

### Stage two: Identifying relevant studies

The search strategy will aim to locate both published and unpublished sources. As recommended in the JBI guidelines, a three-step search strategy will be used. First, a broad search of databases (including EMBASE, Medline (OVID), and CINAHL), using keywords for cancer, education, employment, rehabilitation, adolescents and young adults will be completed. Second, words contained in the titles and abstracts of relevant manuscripts and index terms used to describe these will be used to inform a full search strategy. The medical librarian (DM) will develop a search strategy in collaboration with the lead author (NA) ( Appendix 1). The search strategy will be adapted for each database and information source. Databases that will be searched as part of this scoping review include EMBASE, Web of Science, Medline (OVID), CINAHL, and PsycInfo. Google Scholar will be searched for grey literature, limited to the first 100 searches (e.g., organisation and institutional reports, theses and dissertations, conference materials, programme descriptions/evaluations, etc.). Third, the reference list of all included sources of evidence will be screened for additional studies. The searches will be conducted by NA and DM. Studies published in English will be included, as the resources for translation are not available and no limitation will be set on date. In addition, the lead author will consult the research steering committee, of whom all are key AYA stakeholders, regarding their knowledge on any additional grey literature. The definitive search strategy and results will be reported in detail in the published scoping review.

### Stage three: Selecting studies

Following the search, all identified citations will be collated and uploaded to Covidence and duplicates removed. Prior to screening, two independent reviewers will complete pilot testing of eligibility criteria. A sample of 25 titles/abstracts selected at random will be selected and screened. The team will meet to discuss any discrepancies and amend eligibility criteria as needed. Screening will commence when at least 75% agreement is achieved ( Peters *et al.*, 2020). Titles and abstracts will be screened by the first author for assessment against inclusion criteria. The full texts of selected studies will then be reviewed for inclusion by two reviewers. Where uncertainty occurs, a third reviewer will be consulted and resolved through discussion. Full text studies will be excluded if they do not meet the inclusion criteria and reasons for exclusion recorded. The search results will be reported in a PRISMA flow diagram ( Page *et al.*, 2021).

### Step four: Charting the data

Data extraction will focus on identifying and charting data relating to interventions that support education and/or employment outcomes for AYAs following a cancer diagnosis. A data charting form will be developed using Microsoft Excel. This will be developed using the JBI data extraction tool and will be guided by the Intervention Development and Replication (TIDieR) checklist ( Hoffmann *et al.*, 2014) ( Appendix 2), which systematically describes interventions and their characteristics to support future replication. Two reviewers will pilot the data charting form by independently extracting data from the first ten included studies. They will then meet to confirm consistency in their approach in data extraction as well as establishing suitability of the data charting form. The form may be subject to amendment as familiarity with selected studies necessitates a need for capturing further information. Where uncertainty occurs, a third reviewer will be consulted and resolved through discussion.

The data extraction tool will collect the following data related to included studies/resources:

1. Author
2. Title
3. Year of publication
4. Evidence source details
5. Country of origin
6. Study/programme aims/objectives/purpose
7. Research design (if applicable)
8. Inclusion/exclusion criteria
9. Demographic details of participants included (if applicable) (e.g., cancer type, age)
10. Sample size (if applicable)
11. Intervention format (online, in-person, written)
12. Frequency of intervention delivery (once-off, six-weeks, etc.)
13. Intervention duration (e.g., one-hour/session, 30 minutes etc.)
14. Intervention content
15. Intervention facilitator(s) (if applicable)
16. Theoretical framework (if applicable)
17. Outcomes measured (if applicable)
18. Comparator (if applicable)
19. Caregiver/Partner involvement (if applicable)

Data will be extracted by the lead author and verified by a second. Our objective is to map the existing literature rather than assess the methodological quality of included studies. Therefore, we do not plan to conduct a formal quality appraisal. This approach is consistent with scoping review guidance, which considers quality assessment optional.

### Step five: Collating, summarising, analysing and presenting the data

The PRISMA-ScR checklist will be used to guide the reporting of this review ( Tricco *et al*., 2018). Literature search findings and the study screening process will be presented in a PRISMA flow diagram. Extracted data will be charted in tabular format using the TIDieR checklist to ensure comprehensive and consistent reporting of intervention characteristics. Following data charting, findings will be synthesised using both a descriptive narrative summary and the PAGER framework (Patterns, Advances, Gaps, Evidence for practice, and Research recommendations) ( Bradbury-Jones *et al*., 2022). PAGER will provide a structured approach to analysing and presenting the results, enabling clearer identification of key themes, developments in the field, gaps in the evidence base, and implications for research and practice. This will ensure that the synthesis aligns with and supports the scoping review’s objectives and questions. Any deviation from the protocol will be clearly explained in the complete scoping review report.

## Discussion

Globally the number of newly diagnosed AYA cancers is increasing and so too is the number of AYA living with and beyond cancer *.* Physical and psychosocial sequalae of cancer and its treatment can impact on AYAs reintegrating back to education and/or employment following cancer treatment. A gap in current research includes education and employment reintegration, where AYAs self-identified research on return to education and work as a top priority in a UK research priority-setting exercise ( Aldiss *et al.*, 2019). Therefore, the exploration of factors impacting on successful return to education and employment for AYA cancer survivors is warranted.

To our knowledge, this scoping review will be the first to focus on interventions that support return to education and/or employment for AYAs following a cancer diagnosis. This protocol will include a comprehensive search of peer reviewed and grey literature and will identify, map and summarise existing literature describing supports programmes for AYAs that measure an education or employment outcome.

## Ethical considerations

As scoping review methodology involves reviewing and collecting data from publicly accessible material, this study does not require ethical approval.

## Ethics and consent

Ethical approval and consent were not required.

## Figures and Tables

**Table:** 

Item Number	Item	Where located?	
		Primary paper (page or appendix number)	Other details
1	**Brief name** *(Name of phrase that describes the interventions)*		
2	**Why** *(Rationale, theory, or goal of the elements essential to the intervention)*		
3	**What** *(Describe any physical of informational materials used in the intervention and how they were provided)*		
4	**Procedures** *(Describe each procedure, activity, and/or process used in the intervention)*		
5	**Who provided** *(For each provider, describe their expertise, background, and any specific training given)*		
6	**How** *(Describe modes of delivery)*		
7	**Where** *(Describe type(s) of location(s) where the intervention occurred)*		
8	**When and how much** *(Describe the number of times the intervention was delivered and over what period of time)*		
9	**Tailoring** *(If the intervention was planned to be personalised, titrated, or adapted, then describe what, why, when, and how)*		
10	**Modifications** *(Describe any changes during the course of the study/ intervention)*		
11	**Planned** *(If intervention adherence or fidelity was assessed, describe how and by whom; if any strategies were used to maintain or improve fidelity, describe them.)*		
12	**Actual** *(If intervention adherence or fidelity was assessed, describe the extent to which the intervention was delivered as planned.)*		

## Data Availability

No data are associated with this article.

## Appendix 1: Search strategy

Non-pharmacological interventions to support education and/or work outcomes for adolescents and young adults (AYAs) with cancer.

### EMBASE

'neoplasm'/exp OR 'cancer rehabilitation'/exp OR 'cancer patient'/exp

(cancer* or neoplas* or tumor* or tumour* or malignan* or carcinoma* or metasta* or oncolog* or leukemi* or leukaemi* or lymphoma* or myeloma* or sarcoma*):ti,ab,kw

#1 OR #2

'cancer survival'/exp OR 'cancer survivor'/exp OR 'childhood cancer survivor'/exp OR 'survivorship'/exp

Surviv*:ti,ab,kw

#4 OR #5

'juvenile'/de OR 'adolescent'/de OR 'adolescent health'/exp OR 'child'/exp OR 'young adult'/exp

(Child OR childhood OR children OR infant* OR baby OR babies OR newborn OR preschool OR 'pre school' OR school OR ‘high school’ OR teenager* OR teen? OR adolescen* OR juvenile* OR ‘young adult*’ OR ‘young cancer patient*’ OR ‘young cancer survivor*’ OR ‘young patient*’):ti,ab,kw

'pediatrics'/exp

p?ediatric*:ti,ab,kw,jt

#7 OR #8 OR #9 OR #10

'outcome assessment'/exp AND ('employment'/exp OR 'unemployment'/exp)

'vocational guidance'/exp

((educat* OR work OR employ* OR vocation* OR school OR university OR occupational) NEAR/3 (outcome* OR support* OR intervention* OR needs OR program* OR lnclusion)):ti,ab,kw

#12 OR #13 OR #14

#3 AND #6 AND #11 AND #15

### Medline

exp Neoplasms/

(cancer* or neoplas* or tumor* or tumour* or malignan* or carcinoma* or metasta* or oncolog* or leukemi* or leukaemi* or lymphoma* or myeloma* or sarcoma*).mp.

1 OR 2

Cancer Survivors/ OR Survivors/

Surviv*.mp.

4 OR 5

exp adolescent/ or exp child/ or exp infant/ or (infant disease* or childhood disease*).ti,ab,kf. or (adolescen* or babies or baby or boy? or boyfriend or boyhood or girlfriend or girlhood or child* or girl? or infan* or juvenil* or kid? or minors or minors* or neonat* or neo-nat* or newborn* or new-born* or paediatric* or peadiatric* or pediatric* or perinat* or preschool* or puber* or pubescen* or school* or teen* or toddler? or underage? or under-age? or youth*).ti,ab,kf. or (pediatric* paediatric* or infan* or child* or adolescen* or young).jn,jw. or (pediatric* or paediatric* or infan* or child* or adolescen* or young).in.

(exp Rehabilitation, Vocational/ OR exp Employment/) AND Treatment Outcome/

((educat* OR work OR employ* OR vocation* OR school OR university OR occupational) adj3 (outcome* OR support* OR intervention* OR needs OR program* OR inclusion)).mp.

8 OR 9

### CINAHL

(MH "Childhood Neoplasms") OR (MH "Neoplasms")

TI (cancer* or neoplas* or tumor* or tumour* or malignan* or carcinoma* or metasta* or oncolog* or leukemi* or leukaemi* or lymphoma* or myeloma* or sarcoma*) OR AB (cancer* or neoplas* or tumor* or tumour* or malignan* or carcinoma* or metasta* or oncolog* or leukemi* or leukaemi* or lymphoma* or myeloma* or sarcoma*)

S1 OR S2

(MH "Cancer Survivors") OR (MH "Survivors")

TI (survivor*) OR AB (survivor*)

S4 OR S5

(MH "Young Adult") OR (MH "Adolescence+") OR (MH "Child+") OR (MH "Students, High School") OR (MH "Students, Middle School") OR (MH "Schools, Secondary") OR (MH "Schools, Middle") OR (MH "Schools, Elementary")

TI (Child OR childhood OR children OR infant* OR baby OR babies OR newborn OR preschool OR “pre school” OR school OR “high school” OR teenager* OR teen* OR adolescen* OR juvenile* OR “young adult*” OR “young cancer patient*” OR “young cancer survivor*” OR “young patient*”) OR AB (Child OR childhood OR children OR infant* OR baby OR babies OR newborn OR preschool OR “pre school” OR school OR “high school” OR teenager* OR teen* OR adolescen* OR juvenile* OR “young adult*” OR “young cancer patient*” OR “young cancer survivor*” OR “young patient*”)

(MH "Pediatrics+")

TI (P#ediatrics) OR AB (P#ediatrics)

S7 OR S8 OR S9 OR S10

(MH "Educational Status") OR (MH "Employment Status") OR (MH "Outcomes of Education") OR (MH "Unemployment")

TI ((educat* OR work OR employ* OR vocation* OR school OR university OR occupational) N2 (outcome* OR support* OR intervention* OR needs OR program* OR inclusion)) OR AB ((educat* OR work OR employ* OR vocation* OR school OR university OR occupational) N2 (outcome* OR support* OR intervention* OR needs OR program* OR inclusion))

S12 OR S13

### PsycINFO

DE "Neoplasms" OR DE "Benign Neoplasms" OR DE "Breast Neoplasms" OR DE "Endocrine Neoplasms" OR DE "Leukemias" OR DE "Melanoma" OR DE "Metastasis" OR DE "Nervous System Neoplasms" OR DE "Terminal Cancer"

TI (cancer* or neoplas* or tumor* or tumour* or malignan* or carcinoma* or metasta* or oncolog* or leukemi* or leukaemi* or lymphoma* or myeloma* or sarcoma*) OR AB (cancer* or neoplas* or tumor* or tumour* or malignan* or carcinoma* or metasta* or oncolog* or leukemi* or leukaemi* or lymphoma* or myeloma* or sarcoma*)

S1 OR S2

DE "Survivors"

TI (survivor*) OR AB (survivor*)

S4 OR S5

TI (Child OR childhood OR children OR infant* OR baby OR babies OR newborn OR preschool OR “pre school” OR school OR “high school” OR teenager* OR teen* OR adolescen* OR juvenile* OR “young adult*” OR “young cancer patient*” OR “young cancer survivor*” OR “young patient*”) OR AB (Child OR childhood OR children OR infant* OR baby OR babies OR newborn OR preschool OR “pre school” OR school OR “high school” OR teenager* OR teen* OR adolescen* OR juvenile* OR “young adult*” OR “young cancer patient*” OR “young cancer survivor*” OR “young patient*”)

TI (P#ediatrics) OR AB (P#ediatrics)

S7 OR S8

TI ((educat* OR work OR employ* OR vocation* OR school OR university OR occupational) N2 (outcome* OR support* OR intervention* OR needs OR program* OR inclusion)) OR AB ((educat* OR work OR employ* OR vocation* OR school OR university OR occupational) N2 (outcome* OR support* OR intervention* OR needs OR program* OR inclusion))

### Web of science core collection

(cancer* or neoplas* or tumor* or tumour* or malignan* or carcinoma* or metasta* or oncolog* or leukemi* or leukaemi* or lymphoma* or myeloma* or sarcoma*)

Surviv*

(Child OR childhood OR children OR infant* OR baby OR babies OR newborn OR preschool OR "pre school" OR school* OR "high school" OR teenager* OR teen* OR adolescen* OR juvenile* OR "young adult*" OR "young cancer patient*" OR "young cancer survivor*" OR "young patient*" OR pediatric* OR paediatric*)

((educat* OR work OR employ* OR vocation* OR school OR university OR occupational) NEAR/3 (outcome* OR support* OR intervention* OR needs OR program* OR inclusion))

#1 AND #2 AND #3 AND #4

### Google scholar

Cancer|tumor|tumour|neoplasm survivor|survival adolescent|adolescents|”young adults”|AYA “education|educational|work|employment|vocational|school|university|occupational AROUND(3) outcome|support|intervention|needs|programme|programme”
