# Supplementary material for: Non-pharmacological interventions to support return to education and work for adolescent and young adults (AYAs) following a cancer diagnosis: a scoping review protocol
Source: HRB Open Res. 2026 Jan 16;8:44. Originally published 2025 Mar 24. [Version 2] doi: 10.12688/hrbopenres.14096.2 (PMC12848347; doi:10.12688/hrbopenres.14096.2)
Supplement: Peer review history

# Reviewer report: Reviewer response for version 2

**DOI:** 10.21956/hrbopenres.15768.r52902

**Published:** 2026-01-27

**Authors:** Meghan E. McGrady

**Affiliations:** 1 Cincinnati Children's Hospital Medical Center, Cincinnati, USA

The authors have provided clear and thoughtful responses to my prior comments and I have no remaining questions.

Is the study design appropriate for the research question?

Partly

Is the rationale for, and objectives of, the study clearly described?

Partly

Are sufficient details of the methods provided to allow replication by others?

Partly

Are the datasets clearly presented in a useable and accessible format?

Not applicable

Reviewer Expertise:

adolescent and young adult psycho-oncology

I confirm that I have read this submission and believe that I have an appropriate level of expertise to confirm that it is of an acceptable scientific standard.

# Reviewer report: Reviewer response for version 2

**DOI:** 10.21956/hrbopenres.15768.r52901

**Published:** 2026-01-19

**Authors:** Suja Somanadhan

**Affiliations:** 1 University College Dublin, Dublin, Leinster, Ireland

Thank you for the opportunity to review the updated version of this review protocol. The protocol reads very well, and I thank the authors for addressing all of my comments.

 I have only a minor edit regarding the keywords mentioned in the response, which do not appear to have been updated in Version 2. 

 Best Wishes

 Suja

Is the study design appropriate for the research question?

Yes

Is the rationale for, and objectives of, the study clearly described?

Yes

Are sufficient details of the methods provided to allow replication by others?

Partly

Are the datasets clearly presented in a useable and accessible format?

Not applicable

Reviewer Expertise:

Child and family health, Rare diseases, Transition, Psychosocial Research, Outcome Measures

I confirm that I have read this submission and believe that I have an appropriate level of expertise to confirm that it is of an acceptable scientific standard.

# Reviewer report: Reviewer response for version 1

**DOI:** 10.21956/hrbopenres.15487.r50280

**Published:** 2025-10-10

**Authors:** Meghan E. McGrady, Jennifer Kelleher

**Affiliations:** 1 Cincinnati Children's Hospital Medical Center, Cincinnati, Ohio, USA

**Study Rationale and Objectives**

 The authors clearly outline the impacts of cancer and its treatment on educational and occupational outcomes, highlighting the need to identify interventions with the potential to support AYAs in these domains. While the rationale for the larger study is clear, there is an opportunity to clarify the types of interventions being targeted in this review. Specifically, the authors use multiple terms to describe the types of interventions including “non-pharmacological,” “rehabilitation”, and “support programmes.” While the definition in the “concept” section is clear, the interchangeable use of the aforementioned terms leads to some confusion. For example, the phrase “rehabilitation interventions and support programmes” is used in the “population” section, raising a question as to whether other intervention types (e.g., psychoeducation intervention supporting return to work) will be included. In addition, the use of the term “non-pharmacological” introduces a question as to whether there are pharmacological interventions that target these outcomes and if so, why they are excluded. The authors may wish to consider whether it could be helpful to include the definition in the “concept” section earlier in the manuscript and thereafter simply refer to them as “interventions” thereafter to promote consistency.

**Study Design**

 The study design and methods are appropriate to the research question and rigor is enhanced by adherence to standardized guidelines. The research questions, however, do cover a broad range of topics, and the authors may wish to consider clarifying primary (versus secondary) aims.

 In addition, the authors may wish to consider the following points:

 1. It is somewhat unclear why cross-sectional studies could be included in this review. The authors may wish to consider if/why/how data from these studies would be appropriate for answering the stated research questions.

 2. The authors may wish to consider whether it would be helpful to expand their planned data extraction to include the following elements: comparator condition, caregiver/care partner involvement (e.g., is this an AYA-focused intervention or is a caregiver also involved and if so, in what capacity), target outcome of the intervention (as studies may measure multiple outcomes in addition to the intervention target)

 3. Since many studies include multiple outcomes, the authors may wish to consider a framework for identifying the “primary” outcome of each study, or the outcome which will be used as the authors “summarized the reported impact” of these interventions.

**Methods**

 The methods section is generally clear. The authors may wish to consider the following points to ensure sufficient detail is provided to allow for replication:

 1. While an age range of 15-39 years is stated, it is unclear if this refers to the age at diagnosis or age at enrollment in the intervention.

 2. As mentioned by the authors, as there are multiple definitions of the AYA age range, the authors may wish to consider and articulate if they will include interventions that include AYAs spanning part of this age range. For example, will they only include interventions whose eligible age range falls within 15-39 years (thus excluding a study that could focus on ages 12-24) or will they include interventions in which the majority of the sample or mean age of the sample falls within 15-39 years (and thus could include the prior example)? Or will they use some other criteria?

 3. As the review seems to focus on a broad range of interventions as long as they target education and/or employment outcomes, it may be helpful to include details on what measures would be considered markers of these outcomes.

 4. Could the authors provide a definition of types of “grey literature” that could be included?

Is the study design appropriate for the research question?

Partly

Is the rationale for, and objectives of, the study clearly described?

Partly

Are sufficient details of the methods provided to allow replication by others?

Partly

Are the datasets clearly presented in a useable and accessible format?

Not applicable

Reviewer Expertise:

AYA psycho-oncology

We confirm that we have read this submission and believe that we have an appropriate level of expertise to confirm that it is of an acceptable scientific standard, however we have significant reservations, as outlined above.

# Response

**Published:** 2025-12-23

**Authors:** Naomi Algeo

**Affiliations:** Occupational Therapy, Trinity College Dublin School of Medicine, Dublin, Leinster, Ireland

Dear Reviewers, 

 Many thanks for your feedback on our scoping review protocol. Please find below our responses in how we have addressed your helpful feedback:

***(1) The authors clearly outline the impacts of cancer and its treatment on educational and occupational outcomes, highlighting the need to identify interventions with the potential to support AYAs in these domains. While the rationale for the larger study is clear, there is an opportunity to clarify the types of interventions being targeted in this review. Specifically, the authors use multiple terms to describe the types of interventions including “non-pharmacological,” “rehabilitation”, and “support programmes.” While the definition in the “concept” section is clear, the interchangeable use of the aforementioned terms leads to some confusion. For example, the phrase “rehabilitation interventions and support programmes” is used in the “population” section, raising a question as to whether other intervention types (e.g., psychoeducation intervention supporting return to work) will be included. In addition, the use of the term “non-pharmacological” introduces a question as to whether there are pharmacological interventions that target these outcomes and if so, why they are excluded. The authors may wish to consider whether it could be helpful to include the definition in the “concept” section earlier in the manuscript and thereafter simply refer to them as “interventions” thereafter to promote consistency.***

 RESPONSE:

 Thank you for this comment. We acknowledge that the use of multiple terms to describe interventions may have caused confusion. To clarify, we intentionally use the term “non-pharmacological interventions” because there is existing literature, including medical and pharmacological studies (e.g., de Boer et al., 2015 Cochrane review), that examine the effects of pharmacological approaches on employment outcomes in cancer. By specifying “non-pharmacological” we clearly delineate the scope of our review to behavioural, psychosocial, vocational, educational, and rehabilitation-oriented strategies.

 To improve clarity and consistency, we will introduce this definition early in the manuscript and, thereafter, refer to these approaches simply as “interventions”, ensuring consistent terminology while maintaining the precision needed to define the scope of included studies. We have clarified this in the Methods section:

*Concept:* The proposed scoping review is designed to explore non-pharmacological interventions that support education and/or employment outcomes for AYAs. Interventions can be group-based, individual, and/or online in format, and can be vocational, psychosocial, physical, educational, or multidisciplinary in nature. Pharmacological or medical interventions are excluded, as the focus of this review is on behavioural, psychosocial, vocational, educational, and rehabilitation-oriented strategies. From here on in, we will refer to these approaches simply as interventions.

***(2) The study design and methods are appropriate to the research question and rigor is enhanced by adherence to standardized guidelines. The research questions, however, do cover a broad range of topics, and the authors may wish to consider clarifying primary (versus secondary) aims.***

 RESPONSE: 

 Thank you – we have made a slight amendment to clarify the primary aims and then secondary objectives:

 The primary aim of this scoping review is to identify and summarise the core elements (i.e., content, delivery, resources, length, and format) of non-pharmacological interventions/supports for adolescents and young adults who have had a cancer diagnosis to support return to education and/or work.

 Secondary objectives include:…

***(3) In addition, the authors may wish to consider the following points:***

***1. It is somewhat unclear why cross-sectional studies could be included in this review. The authors may wish to consider if/why/how data from these studies would be appropriate for answering the stated research questions.***

 RESPONSE:

 Thank you for this comment. We agree that the inclusion of cross-sectional studies requires clarification. Cross-sectional studies will be included in this scoping review because they can provide valuable descriptive information on the types of interventions and outcomes being assessed, even if they do not evaluate change over time. These data will contribute to mapping the landscape of available interventions and reported outcomes, complementing longitudinal and experimental studies that assess intervention impact. We will clearly indicate study design in our data extraction and reporting to ensure transparency. We have added:

 While some study designs (e.g., cross-sectional, qualitative, etc.) do not allow assessment of intervention effectiveness over time, they may provide valuable information on the types of interventions offered to AYAs and the range of outcomes measured. Therefore, we will include these studies to capture descriptive data on interventions and outcomes.

***(4) The authors may wish to consider whether it would be helpful to expand their planned data extraction to include the following elements: comparator condition, caregiver/care partner involvement (e.g., is this an AYA-focused intervention or is a caregiver also involved and if so, in what capacity), target outcome of the intervention (as studies may measure multiple outcomes in addition to the intervention target)***

 RESPONSE:

 Thank you – we have added the below to the data extraction:

 The data extraction tool will collect the following data related to included studies/resources:

1. Author
2. Title
3. Year of publication
4. Evidence source details
5. Country of origin
6. Study/programme aims/objectives/purpose
7. Research design (if applicable)
8. Inclusion/exclusion criteria
9. Demographic details of participants included (if applicable) (e.g., cancer type, age)
10. Sample size (if applicable)
11. Intervention format (online, in-person, written)
12. Frequency of intervention delivery (once-off, six-weeks, etc.)
13. Intervention duration (e.g., one-hour/session, 30 minutes etc.)
14. Intervention content
15. Intervention facilitator(s) (if applicable)
16. Theoretical framework (if applicable)
17. Outcomes measured (if applicable)
18. Comparator (if applicable)
19. Caregiver/Partner involvement (if applicable)

***(5) Since many studies include multiple outcomes, the authors may wish to consider a framework for identifying the “primary” outcome of each study, or the outcome which will be used as the authors “summarized the reported impact” of these interventions.***

 RESPONSE

 Thank you for this helpful suggestion. We recognize that many studies report multiple outcomes. In this scoping review, we will extract all outcomes relevant to education and/or employment and summarise the impact of interventions based on those outcomes. When multiple outcomes are reported, we will describe the range of impacts and highlight those most directly aligned with the review objectives. This approach is consistent with scoping review methodology, which focuses on mapping and describing the breadth of evidence rather than identifying a single primary outcome.

 We appreciate that a rewording of the objective may be useful for clarity, and have added:

 ‘To identify and describe the range of outcomes reported for non-pharmacological interventions and support programmes/services that support return to education and/or employment for AYAs.’

***(6) The methods section is generally clear. The authors may wish to consider the following points to ensure sufficient detail is provided to allow for replication:***

***1. While an age range of 15-39 years is stated, it is unclear if this refers to the age at diagnosis or age at enrolment in the intervention.***

 RESPONSE:

 Thank you – this has been clarified throughout the text to confirm that we are considering AYAs who were diagnosed with cancer between 15-39 years.

***(7) As mentioned by the authors, as there are multiple definitions of the AYA age range, the authors may wish to consider and articulate if they will include interventions that include AYAs spanning part of this age range. For example, will they only include interventions whose eligible age range falls within 15-39 years (thus excluding a study that could focus on ages 12-24) or will they include interventions in which the majority of the sample or mean age of the sample falls within 15-39 years (and thus could include the prior example)? Or will they use some other criteria?***

 RESPONSE:

 Thank you – we have clarified this in the text under Stage One section: ‘Intervention eligibility will only be considered if age range falls within 15-39 years (e.g. study focusing on ages 12-24 would be excluded).’

***(8) As the review seems to focus on a broad range of interventions as long as they target education and/or employment outcomes, it may be helpful to include details on what measures would be considered markers of these outcomes.***

 RESPONSE

 Thank you – we have included some examples of how these outcomes could be interpreted. For example, in Table 1 we have added:  ‘Outcomes may include school reintegration (e.g., e.g., binary yes/no for education status, academic performance, etc.), return-to-work (e.g., binary yes/no for working status; work satisfactions etc.), or employability.’

***(9) Could the authors provide a definition of types of “grey literature” that could be included?***

 RESPONSE

 Thank you – we have included examples of grey literature to be considered: ‘(e.g., organisation and institutional reports, theses and dissertations, conference materials, programme descriptions/evaluations, etc.).’.

# Reviewer report: Reviewer response for version 1

**DOI:** 10.21956/hrbopenres.15487.r47030

**Published:** 2025-05-27

**Authors:** Suja Somanadhan

**Affiliations:** 1 University College Dublin, Dublin, Leinster, Ireland

Thank you for the opportunity to review this scoping review protocol. This is a strong and well-structured paper with clear relevance to the field. However, I have highlighted several areas where further clarification or refinement could strengthen the overall quality and rigour of the protocol. Addressing these points will enhance your work's transparency, methodological robustness, and impact.

 The listed keywords—cancer survivorship, employment, quality of life, reintegration, schooling—require revision to reflect the review's scope and content. For instance, the table of contents includes themes such as adolescents and young adults and non-pharmacological interventions, which are not currently captured in the keywords. Please consider aligning keywords with major review concepts to enhance discoverability.

 Inclusion and Exclusion Criteria lack clarity in their current narrative form. Presenting these in a tabular format would improve transparency and make it easier for readers to understand the eligibility parameters.

 Search Strategy: Please provide a sample search strategy in a table format, including databases, search terms, and limits used. This is important for reproducibility and methodological rigour.

 Quality Appraisal: It is unclear whether a quality appraisal will be conducted as part of this scoping review. Please clarify this. If it is not planned, provide a rationale explaining why, it is in line with scoping review methodology.

 PPI and Knowledge User Engagement: Please specify whether the review will involve consultation or engagement with Patient and Public Involvement (PPI) representatives or other knowledge users. This is increasingly considered a valuable aspect of comprehensive and relevant reviews.

 Stage 5—Reporting of Data: The approach to data reporting needs greater clarity. Specifically, will the PAGER framework (Patterns, Advances, Gaps, Evidence for practice, and Research recommendations) be used? This framework (Bradbury-Jones et al., 2022) provides a systematic method for presenting scoping review findings, thereby enhancing the interpretability and utility of the results.

Is the study design appropriate for the research question?

Yes

Is the rationale for, and objectives of, the study clearly described?

Yes

Are sufficient details of the methods provided to allow replication by others?

Partly

Are the datasets clearly presented in a useable and accessible format?

Not applicable

Reviewer Expertise:

Child and family health, Rare diseases, Transition, Psychosocial Research, Outcome Measures

I confirm that I have read this submission and believe that I have an appropriate level of expertise to confirm that it is of an acceptable scientific standard, however I have significant reservations, as outlined above.

# Response

**Published:** 2025-12-23

**Authors:** Naomi Algeo

**Affiliations:** Occupational Therapy, Trinity College Dublin School of Medicine, Dublin, Leinster, Ireland

Dear Reviewer One, Many thanks for your manuscript feedback. Please see below for clarity in how we have addressed each point of feedback:

***(1) 'The listed keywords—cancer survivorship, employment, quality of life, reintegration, schooling—require revision to reflect the review's scope and content. For instance, the table of contents includes themes such as adolescents and young adults and non-pharmacological interventions, which are not currently captured in the keywords. Please consider aligning keywords with major review concepts to enhance discoverability.'***

 RESPONSE:

 Thank you for this suggestion. While we recognise that key concepts appear in the title, we agree that including these terms in the keyword list will enhance discoverability and ensure that all major components of the review, particularly adolescents and young adults (AYAs), non-pharmacological interventions, and return to education and work, are clearly indexed. We have therefore updated the keyword list to better reflect the scope and content of the review, while acknowledging that there will be terms within the title that also enhance discoverability.

 Keywords: cancer survivorship; employment; psychosocial support; reintegration; young adult oncology; vocational rehabilitation.

***(2) Inclusion and Exclusion Criteria lack clarity in their current narrative form. Presenting these in a tabular format would improve transparency and make it easier for readers to understand the eligibility parameters.***

 RESPONSE:

 Thank you for your comment. We have added a table to clearly label and categorise eligibility criteria that aligns with JBI scoping review standards/PCC framework.  

***(3) Search Strategy: Please provide a sample search strategy in a table format, including databases, search terms, and limits used. This is important for reproducibility and methodological rigour.***

 RESPONSE: 

 Thank you. We have added a sample search strategy as an appendix.

***(4) Quality Appraisal: It is unclear whether a quality appraisal will be conducted as part of this scoping review. Please clarify this. If it is not planned, provide a rationale explaining why, it is in line with scoping review methodology.***

 RESPONSE:

 Following established scoping review methodology (JBI), our objective is to map the existing literature rather than assess the methodological quality of included studies. Therefore, we do not plan to conduct a formal quality appraisal. This approach is consistent with scoping review guidance, which considers quality assessment optional. We have outlined this in the manuscript.

 ‘Our objective is to map the existing literature rather than assess the methodological quality of included studies. Therefore, we do not plan to conduct a formal quality appraisal. This approach is consistent with scoping review guidance, which considers quality assessment optional.’

***(5) PPI and Knowledge User Engagement: Please specify whether the review will involve consultation or engagement with Patient and Public Involvement (PPI) representatives or other knowledge users. This is increasingly considered a valuable aspect of comprehensive and relevant reviews.***

 RESPONSE

 Thank you – Yes, we are delighted that we had one PPI member of our research team who directly influenced the conception of this protocol. We have added:

*‘Patient and Public Involvement: *A patient and public involvement (PPI) contributor, who is also a co-author on this protocol (SB), was involved in the development of the review objectives, refinement of the research question, and identification of key concepts relevant to AYAs following a cancer diagnosis. The PPI member provided feedback on the scope of the review, the relevance of outcomes relating to education and employment, and contributed to ensuring that the planned review reflects patient priorities. No additional consultation stage is planned beyond their ongoing involvement in interpreting findings during the analysis and dissemination stages.’

***(6) Stage 5—Reporting of Data: The approach to data reporting needs greater clarity. Specifically, will the PAGER framework (Patterns, Advances, Gaps, Evidence for practice, and Research recommendations) be used? This framework (Bradbury-Jones et al., 2022) provides a systematic method for presenting scoping review findings, thereby enhancing the interpretability and utility of the results.***

 RESPONSE:

 Thank you for this helpful suggestion. We agree that greater clarity around our approach to reporting the data is needed. We will extract intervention-related information using the TIDieR checklist, which provides a comprehensive and structured method for capturing key characteristics and components of the interventions included in the review. To support transparent and meaningful synthesis at Stage 5, we will also use the PAGER framework as outlined by Bradbury-Jones et al. (2022). Together, TIDieR and PAGER will provide both the structured extraction of intervention details and a coherent approach to synthesising and reporting the review results.

 We have amended as follows:

 Step Five: Collating, summarising, analysing and presenting the data

 The PRISMA-ScR checklist will be used to guide the reporting of this review (Tricco et al., 2018). Literature search findings and the study screening process will be presented in a PRISMA flow diagram. Extracted data will be charted in tabular format using the TIDieR checklist to ensure comprehensive and consistent reporting of intervention characteristics. Following data charting, findings will be synthesised using both a descriptive narrative summary and the PAGER framework (Patterns, Advances, Gaps, Evidence for practice, and Research recommendations) (Bradbury-Jones et al., 2022). PAGER will provide a structured approach to analysing and presenting the results, enabling clearer identification of key themes, developments in the field, gaps in the evidence base, and implications for research and practice. This will ensure that the synthesis aligns with and supports the scoping review’s objectives and questions. Any deviation from the protocol will be clearly explained in the complete scoping review report.
